# Genetic and clinical characteristics of primary and secondary glioblastoma is associated with differential molecular subtype distribution

**DOI:** 10.18632/oncotarget.3440

**Published:** 2015-01-31

**Authors:** Rui Li, Hailin Li, Wei Yan, Pei Yang, Zhaoshi Bao, Chuanbao Zhang, Tao Jiang, Yongping You

**Affiliations:** ^1^ Department of Neurosurgery, The First Affiliated Hospital of Nanjing Medical University, Nanjing, China; ^2^ Beijing Neurosurgical Institute, Capital Medical University, Beijing, China; ^3^ Beijing Institute for Brain Disorders, Brain Tumor Center, Beijing, China; ^4^ Department of Neurosurgery, Beijing Tiantan Hospital, Capital Medical University, Beijing, China

**Keywords:** Primary glioblastomas, Secondary glioblastomas, Molecular subtypes, Whole transcriptome sequencing

## Abstract

Glioblastoma multiforme (GBM) is classified into primary (pGBM) or secondary (sGBM) based on clinical progression. However, there are some limits to this classification for insight into genetically and clinically distinction between pGBM and sGBM. The aim of this study is to characterize pGBM and sGBM associating with differential molecular subtype distribution. Whole transcriptome sequencing data was used to assess the distribution of molecular subtypes and genetic alterations in 88 pGBM and 34 sGBM in a Chinese population-based cohort, and the biological progression and prognostic impact were analyzed by combining clinical information. Forty-one percentage of pGBM were designated as Mesenchymal subtype, while only 15% were the Proneural subtype. However, sGBM displayed the opposite ratio of Mesenchymal (15%) and Proneural (44%) subtypes. Mutations in isocitrate dehydrogenase-1 (IDH1) were found to be highly concentrated in the Proneural subtypes. In addition, patients with sGBM were 10 years younger on average than those with pGBM, and exhibited clinical features of shorter overall survival and frontal lobe tumor location tendency. Furthermore, in sGBM, gene sets related to malignant progression were found to be enriched. Overall, these results reveal the intrinsic distinction between pGBM and sGBM, and provide insight into the genetic and clinical attributes of GBM.

## INTRODUCTION

Glioblastoma multiforme (GBM) is the most lethal type of adult brain tumor, accounting for 60–70% of all gliomas. Despite the advanced treatment, the median survival of patients with GBM is approximately 15 months [1]. Clinically, GBM is divided into primary glioblastoma (pGBM), which progresses rapidly and has an absence of precursor lesions, and secondary glioblastoma (sGBM), which progresses as diffuse astrocytoma (WHO grade II) or anaplastic astrocytoma (WHO grade III) [2-4]. Although pGBM and sGBM display distinct clinical progression, they are histologically indistinguishable. For further insight, research efforts have focused on investigating GBM molecular profiles. Recent studies suggest that isocitrate dehydrogenase-1 (IDH1) mutations, which are frequently detected in sGBM (>80%) but are rare in pGBM (<5%), may be considered as a diagnostic molecular biomarker of sGBM [5-9]. The Cancer Genome Atlas (TCGA) Research Network described a robust gene expression-based molecular classification of GBM into Proneural, Neural, Classical and Mesenchymal subtypes [10].

In the present study, whole transcriptome sequencing data was analyzed to characterize the distribution of molecular subtypes in 88 pGBM and 34 sGBM from a Chinese population-based cohort. Both pGBM and sGBM samples were analyzed for the presence of biomarkers and enriched gene sets. The clinical features of the patients with pGBM and sGBM were assessed, including overall survival time and tumor location. The results suggest that different clinical and genetic profiles of pGBM and sGBM mainly result from the different proportions of the four molecular subtypes in them.

## RESULTS

### Distribution of molecular subtypes and gene alterations in pGBM and sGBM

**Figure 1:**
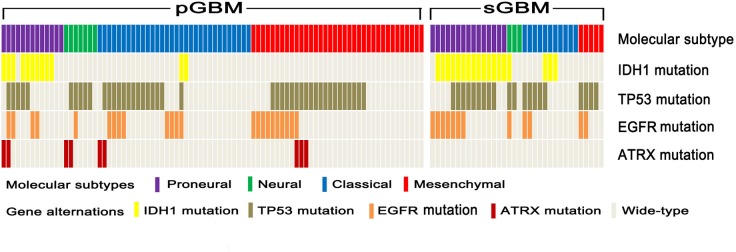
Distribution of molecular subtypes and genetic alteration signatures in pGBM and sGBM Distribution and correlation between GBM molecular subtypes (Proneural, Neural, Classical and Mesenchymal), IDH1 mutation, TP53 mutation, EGFR mutation and ATRX mutation in pGBM and sGBM. Molecular subtypes and genetic alterations are indicated in different colors.

As shown in Figure 1 and Table 1, among 88 pGBM, 36 cases (41%) were Mesenchymal subtype, while only 13 cases (15%) were Proneural subtype. However, sGBM showed the opposite ratio of Mesenchymal (5/34, 15%) and Proneural (15/34, 44%) subtypes. The proportion of Neural subtypes in pGBM (8%) and sGBM (9%) were similar. In addition, 36% of pGBM were classified as Classical subtype, which was slightly higher than 32% of sGBM.

**Table 1:** Clinical features of patients with pGBM and sGBM according to their molecular subtypes

	Proneural		Neural		Classical		Mesenchymal	
	pGBM	sGBM	pGBM	sGBM	pGBM	sGBM	pGBM	sGBM
No. of patients	13	15	7	3	32	11	36	5
Age								
<50 years	10	13	2	0	15	9	12	2
≥50 years	3	2	5	3	17	2	24	3
Gender								
Male	6	13	3	1	20	7	26	3
Female	7	2	4	2	12	4	10	2
Location								
Frontal lobe	5	9	3	3	12	6	9	5
Temporal lobe	6	1	1	0	9	0	14	0

With respect to gene signatures, the frequency of IDH1 mutation in sGBM was 53%, nearly four times as high as that of pGBM (14%). Furthermore, the majority of IDH1 mutations were clustered in Proneural subtypes in both pGBM and sGBM, whereas IDH2 mutation was absent in the whole cohort. TP53 and IDH1 mutations were mutually exclusive in pGBM, however this was not the case in sGBM. Epidermal growth factor receptor (EGFR) mutation was detected in 35% of sGBM and 26% of pGBM. Furthermore, Alpha Thalassemia/Mental Retardation Syndrome X-linked (ATRX) mutation was detected in 10% of pGBM, but was absent in sGBM. Such alteration was mutually exclusive with IDH1 mutation and EGFR mutation, but co-occurred with TP53 mutation in pGBM.

### Gene set enrichment analysis for pGBM and sGBM

**Figure 2:**
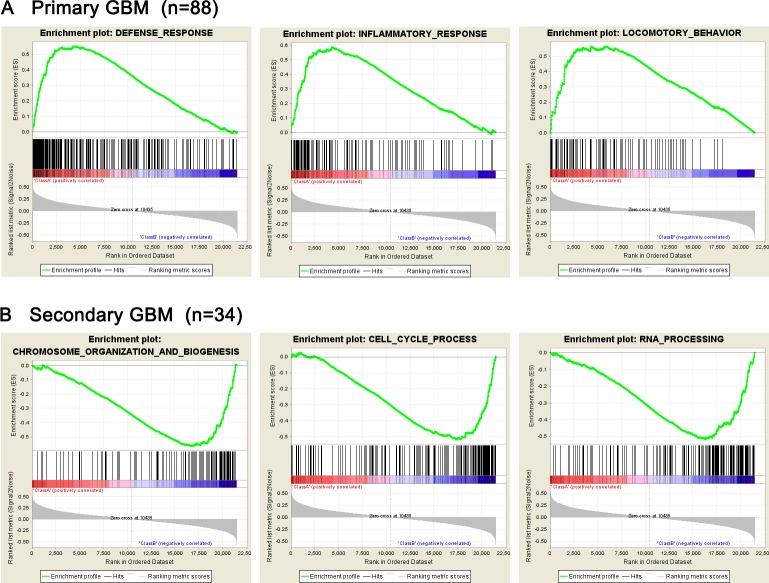
Presence of gene sets related to biological processes analyzed by GSEA (**A**) Gene sets related to biological processes in pGBM; (**B**) Gene sets related to biological processes in sGBM.

Given the data of whole transcriptome sequencing of 88 primary glioblastomas and 34 secondary glioblastomas, we performed Gene set enrichment analysis (GSEA) and got results that gene sets related to defense response, inflammatory response and locomotory behavior were significantly enriched in the primary glioblastomas (P<0.001), while chromosome organization, cell cycle, mRNA processing and mitosis gene sets were clustered in secondary glioblastomas (P<0.001). (Figure 2 and Table S1).

### Prognostic impact of combined analysis of pGBM and sGBM molecular subtypes

**Figure 3:**
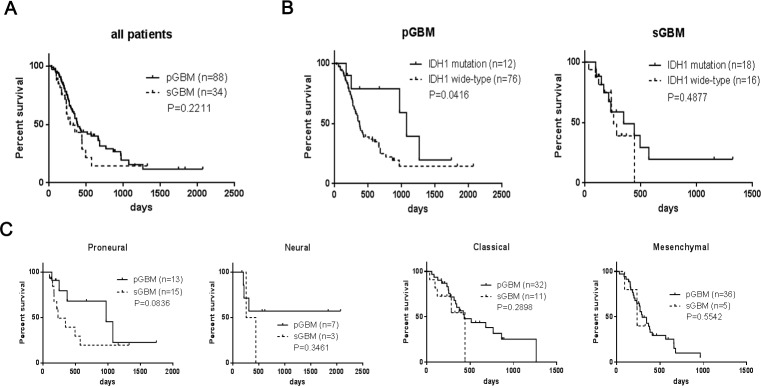
Kaplan–Meier analysis of overall survivals of patients with GBM (**A**) Overall survival of patients with pGBM and sGBM; (**B**) Overall survivals of patients with or without IDH1 mutation in pGBM and sGBM; (C) Overall survivals of patients with Proneural, Neural, Classical and Mesenchymal subtypes.

The median overall survival of all patients with pGBM after diagnosis was 381 days, whereas the median overall survival was 284 days in patients with sGBM (Figure 3A). As shown in (Figure 3B), patients carrying the IDH1 mutation experienced an improved prognosis (1074 days for pGBM and 346 days for sGBM) compared with patients who did not have such a mutation (372 days for pGBM and 256 days for sGBM). When this analysis was combined with molecular subtypes, patients with Neural subtype pGBM exhibited the longest overall survival, followed by patients with Proneural subtype pGBM, with 970 days of median overall survival. Patients with Mesenchymal and Proneural subtypes of sGBM resulted in the worst clinical outcome, with survival of 236 and 231 days, respectively (Figure 3C).

### Clinical features of pGBM and sGBM

**Figure 4:**
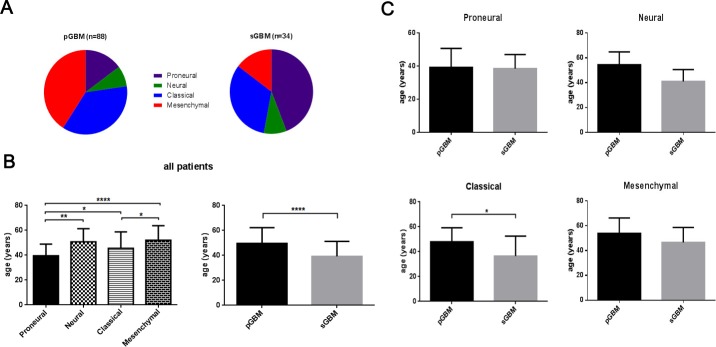
Age distribution of patients with GBM (**A**) Distribution of molecular subtypes in pGBM and sGBM; (**B**) Age distribution of patients with molecular and clinical subtypes of GBM; (**C**) Age distribution of patients with four molecular subtypes of GBM. *, P<0.05; **, P<0.01; ****, P<0.0001.

As shown in (Figure 4), GBM predominantly affected males in this study, with a male to female ratio of 1.67 in pGBM and 2.4 in sGBM. With respect to anatomical localization, the frontal and temporal lobe were the most commonly involved sites, with 68% of sGBM located in the frontal lobe, while only one case involved the temporal lobe. A similar phenomenon was observed in Proneural (45%), Classical (38%) and Neural (58%) subtypes. However, among Mesenchymal subtypes, the temporal lobe was found to be the predominant site (41%). pGBM show widespread anatomical distribution and tumors were more commonly located in the center hemisphere of the brain. The mean age of patients diagnosed with sGBM was 39.26 ± 2.05 years, whereas the mean age of patients diagnosed with pGBM was 49.61 ± 1.35 years. For the molecular subtypes, the cohort with the oldest age of diagnosis was Mesenchymal subtype (52.06 ± 1.64 years), followed by Neural (50.75 ± 3.07 years), Classical (45.50 ± 1.92 years) and Proneural (39.48 ± 1.65 years). The age distribution of the four molecular subtypes in pGBM and sGBM was further analyzed, and patients with Classical subtype sGBM were found to be significantly younger than those with same subtype in pGBM (mean age of 36.45 versus 47.97 years; *P*= 0.0125). This trend was also observed for Mesenchymal subtype (46.60 years for sGBM versus 53.89 for pGBM [*P*=0.2241]), and Neural subtype (41.00 years for sGBM versus 54.43 years for pGBM [*P*=0.0934]), whereas Proneural subtypes had similar age of diagnosis (38.53 years for sGBM versus 39.23 years for pGBM [*P*=0.8552]).

## DISCUSSION

GBM is the most common and lethal type of glioma in adults with an overall survival of less than two years [11-14]. Clinically, GBM is categorized into pGBM and sGBM based on malignant progression. Although they are histologically indistinguishable, pGBM and sGBM can be identified by characterized biomarkers reported in previous studies [7, 11, 15-18].

In the present study, 122 GBM were characterized, including 88 pGBM and 34 sGBM, based on data from whole transcriptome sequencing and clinical information. Each sample was classified into a molecular subtype and found to exhibit different proportions in two histological types of GBM. Approximately 44% of sGBM were classified as Proneural subtypes, which was significantly higher than the proportion of this subtype in pGBM, but not as high as the frequency reported in other studies [8, 10]. In addition, among 15 sGBM with Proneural signature, 14 tumors (93%) carried IDH1 gene mutation. This high frequency of IDH1 mutation was also found in Proneural pGBM, amounting to 77% (10/13), which is higher than the 30% reported in previous studies by Verhaak and colleagues [10] This observation suggests that IDH1 mutation might characterize the Proneural subtype, however this cannot be considered as a definitive marker for sGBM perfectively. From the perspective of molecular subtypes rather than the clinical evaluation, the differences between pGBM and sGBM resulted from the distinct distribution of four molecular subtypes, especially the inverse ratio between Proneural and Mesenchymal subtypes observed in pGBM and sGBM. The distribution of molecular subtypes was also found to impact the survival of patients with pGBM and sGBM. In the cohort analyzed in this study, patients carrying an IDH1 mutation in pGBM exhibited approximately three times longer survival than those without such mutations. However, such advantage of IDH1 mutation on prognostic impact was not observed in sGBM. One would expect that the Mesenchymal subtype, which had the worst prognosis in terms of length of survival, would occupy high frequency in primary glioblastoma.

The ATRX plays an important role in telomere homeostasis via regulating incorporation of histone variant H3.3 into telomeric chromatin [19-21]. ATRX mutations were recently identified in 7% of pGBM and more than half of sGBM and were associated with an alternative lengthening of telomeres (ALT) phenotype among GBM [22, 23]. In the present study, ATRX mutations were detected in 9 of 88 (10%) pGBM, but were absent in sGBM. Furthermore, ATRX mutation co-occurred with TP53 mutation, but was mutually exclusive with IDH1/2 mutation, which is contrary to previous reports [22, 23]. Ethnic and racial disparities, as well as analysis methods may be factors for such differences.

Similar to previous studies, patients diagnosed with sGBM were 10 years younger than patients with pGBM in this cohort [12, 13]. Notably, when analyzed in molecular subtypes, patients with Proneural were 5 years younger than patients with Classical subtypes and 10 years younger than Neural and Mesenchymal subtypes patients. Except for the Classical subtype, there was no significant difference in age between patients with pGBM and sGBM in the three other subtypes. In addition, Proneural and Classical subtypes made up 76% of sGBM patients in this study. These findings verified our hypothesis that different molecular subtype distribution could cause the phenomenon of patients with sGBM being 10 years younger than those with pGBM.

The results from this study suggest that sGBM is predominantly located in the frontal lobe, which is consistent with a previous study by Lai and colleagues [24]. Contrary to sGBM, there was no preferable anatomical cluster location among pGBM. Further combined analysis with whole transcriptome sequencing data revealed that most Neural subtype GBM are located in the frontal lobe, whereas Mesenchymal subtypes are predominantly located in the temporal lobe. Proneural and Classical subtypes were found to be more likely located in the frontal lobe than the temporal lobe. This finding suggests that the predominance of frontal lobe involvement with sGBM partly results from the high frequency of Mesenchymal subtype and rare Proneural subtype in sGBM, which also verified our hypothesis that clinical distinction of pGBM and sGBM glioblastoma was associated with differential molecular subtype distribution.

GSEA was performed for pGBM and sGBM in this study, with enriched gene sets related to inflammatory response, locomotive behavior and defense response found in pGBM, which are critical for protection and progression of tumor cells, while chromosome organization, cell cycle, mRNA processing and mitosis gene sets relating to malignant proliferation of tumor cells were clustered in sGBM. Compared to pGBM, sGBM displayed significant biological progression of malignant transformation and proliferation, which was consistent with the clinical progression of sGBM developed from low-grade glioma.

Overall, these findings demonstrate that the differences between pGBM and sGBM are caused by the molecular subtypes, and highlight the importance of further research into the role of such differences in therapeutic strategies and targeted treatment for pGBM and sGBM.

## MATERIALS AND METHODS

### Tumor samples

A total of 122 GBM samples from the Chinese Glioma Genome Atlas (CGGA) were included in this study, consisting of 88 pGBM and 34 sGBM. Tumor tissue samples were obtained by surgical resection. All pGBM and sGBM cases were defined by two neuropathologists according to the 2007 WHO classification guidelines and Scherer [3]. Only samples with greater than 80% tumor cells were selected. All samples were obtained by surgical resection before radiation and chemotherapy. All patients provided written informed consent, and the study was approved by the ethics committees of the participating hospitals.

### Whole transcriptome sequencing

Whole transcriptome sequencing was performed as described previously [25, 26]. Briefly, total RNA was isolated from disrupted and homogenized frozen tissue samples using the RNeasy Mini Kit (Qiagen) according to the manufacturer's instructions. A pestle and a QIAshredder (Qiagen) were used to disrupt and homogenize frozen tissue. RNA intensity was checked using 2100 Bioanalyzer (Agilent Technologies) and only high quality samples with an RNA Integrity Number (RIN) value greater than or equal to 7.0 were used to construct the sequencing library. The subsequent steps included end repair, adapter ligation, size selection and polymerase chain reaction enrichment. The length of DNA fragment was measured using a 2100 Bioanalyzer, with median insert sizes of 200 nucleotides. The libraries were sequenced on the Illumina HiSeq 2000 platform using the 101-bp pair-end sequencing strategy. Short sequence reads were aligned to the human reference genome (Hg 19 Refseq) using the Burrows-Wheeler Aligner (BWA, Version 0.6.2-r126) SnpEff software was used to annotate genetic variance [27, 28].

### Gene set enrichment analysis

To determine the gene sets related to particular biological processes present in pGBM and sGBM, gene expression profiling and gene set enrichment analysis (GSEA) was performed as described previously [29].

### Statistical analysis

Survival distributions were estimated by Kaplan-Meier survival analysis, and the log-rank test was used to assess the statistical significance between stratified survival groups using GraphPad Prism 5.0 statistical software. Student's t-test was performed using SPSS 13.0. All data are presented as the mean ±SE. A two-sided *P* value < 0.05 was considered significant.

## SUPPLEMENTARY MATERIAL TABLES


