# Supplementary material for: Genetic and clinical characteristics of primary and secondary glioblastoma is associated with differential molecular subtype distribution
Source: Oncotarget. 2015 Jan 31;6(9):7318–24. doi: 10.18632/oncotarget.3440 (PMC4466687; doi:10.18632/oncotarget.3440)
Supplement: Supplementary file 1 [file oncotarget-06-7318-s001.pdf]

Genetic and clinical characteristics of primary and secondary glioblastoma is associated with differential molecular subtype distribution

Supplementary Material

Table S1: Gene sets (TOP 15) enriched in pGBM

	NAME	ES	NES	NOM p-val	FDR q-val
1	DEFENSE_RESPONSE	0.55	2.88	0	0
2	RESPONSE_TO_WOUNDING	0.56	2.78	0	0
3	INFLAMMATORY_RESPONSE	0.59	2.75	0	0
4	RESPONSE_TO_EXTERNAL_STIMULUS	0.49	2.6	0	0
5	IMMUNE_RESPONSE	0.5	2.57	0	0
6	LOCOMOTORY_BEHAVIOR	0.57	2.53	0	0
7	CYTOKINE_METABOLIC_PROCESS	0.66	2.52	0	0
8	CYTOKINE_BIOSYNTHETIC_PROCESS	0.67	2.52	0	0
9	CYTOKINE_PRODUCTION	0.59	2.51	0	0
10	REGULATION_OF_CYTOKINE_BIOSYNTHETIC_PROCESS	0.68	2.5	0	0
11	IMMUNE_SYSTEM_PROCESS	0.46	2.42	0	0
12	POSITIVE_REGULATION_OF_CYTOKINE_BIOSYNTHETIC_PROCESS	0.69	2.34	0	0
13	PROTEIN_SECRETION	0.67	2.34	0	0
14	CYTOKINE_SECRETION	0.79	2.32	0	0
15	POSITIVE_REGULATION_OF_SIGNAL_TRANSDUCTION	0.49	2.27	0	0

Table S2: Gene sets (TOP 15) enriched in sGBM

	NAME	ES	NES	NOM p-val	FDR q-val
1	CHROMOSOME_ORGANIZATION_AND_BIOGENESIS	-0.57	-2.53	0	0
2	CELL_CYCLE_PROCESS	-0.52	-2.51	0	0
3	ESTABLISHMENT_AND_OR_MAINTENANCE_OF_CHROMATIN_ARCHITECTURE	-0.6	-2.5	0	0
4	RNA_PROCESSING	-0.52	-2.47	0	0
5	MRNA_PROCESSING_GO_0006397	-0.6	-2.46	0	0
6	MRNA_METABOLIC_PROCESS	-0.58	-2.46	0	0
7	M_PHASE	-0.55	-2.45	0	0
8	CELL_CYCLE_GO_0007049	-0.47	-2.42	0	0
9	MITOSIS	-0.57	-2.42	0	0
10	M_PHASE_OF_MITOTIC_CELL_CYCLE	-0.56	-2.38	0	0
11	CELL_CYCLE_PHASE	-0.5	-2.37	0	0
12	MITOTIC_CELL_CYCLE	-0.51	-2.36	0	0
13	MICROTUBULE_CYTOSKELETON_ORGANIZATION_AND_BIOGENESIS	-0.67	-2.36	0	0
14	CHROMATIN_ASSEMBLY_OR_DISASSEMBLY	-0.71	-2.35	0	0
15	RNA_SPLICING	-0.54	-2.29	0	0
